# Superior Labral Anterior to Posterior Tear Management in Athletes

**DOI:** 10.2174/1874325001812010303

**Published:** 2018-07-31

**Authors:** Cristin John Mathew, David Mark Lintner

**Affiliations:** Houston Methodist Hospital, Houston, Tx 77030, USA

**Keywords:** Superior Labrum Anterior Posterior tear, SLAP, Overhead Athletes, Baseball Players, SLAP Repair, MRI

## Abstract

**Background:**

The diagnosis and treatment of Superior Labrum Anterior to Posterior (SLAP) tears have been evolving and controversial. The lack of clear diagnostic criteria on physical examination, Magnetic Resonance Imaging (MRI), and arthroscopic evaluation clouds the issue. The high rate of MRI diagnosed SLAP lesions in the asymptomatic population of athletes and non-athletes warrants consideration when planning treatment for those with shoulder pain.

**Objective:**

To provide information on the evaluation, diagnosis and management of SLAP tears in athletes.

**Methods:**

The results of a structured non-operative rehabilitation program are discussed and compared to traditional surgical techniques. The evolution of the author’s treatment algorithm is presented. Results: The successful return to overhand throwing is more common with non-operative treatment than with surgical.

**Conclusion:**

A rehabilitation program focused on stretching the posterior capsule and correcting scapular posture is more successful than surgery for most throwers with SLAP lesions.

## 1 INTRODUCTION

The superior labrum while playing an important role in stability of the shoulder has a high degree of variability in native anatomy, injury pattern, and adaptive anatomy. The location of the long head of the bicep origin has variability as well and can range from anterior to central to posterior locations along the superior rim of the glenoid [1]. The role of the long head of the bicep tendon in shoulder stability is controversial, varying from having just a dynamic role, a passive role or no role at all [2-6] The intra-articular portion of the bicep has been found to be pain generator and can apply tension to the labrum creating labral detachment. Not surprisingly, the treatment of SLAP and bicep anchor lesions is unsettled.

There is increasing evidence that superior labral “Tears”, like meniscal cleavage planes, are frequently present on MRI in asymptomatic overhand throwers. In fact, in the experienced thrower, these “Tears” are now considered adaptive changes by the majority of Major League Baseball team physicians [7]. However, it has been documented that SLAP lesions can cause anterior instability in overhead athletes [3, 8-12]. The presence of a SLAP lesion is thought by other authors to be a primary cause of pain in a thrower’s shoulder due to a posterior-superior shift of humeral rotation creating the internal impingement phenomenon [13-15]. This perceived instability lead to the frequent surgical repair as a cure for either anterior or posterior instability. The endemic rate of MRI visible variations of labral anatomy in asymptomatic overhead throwers should prompt caution before reaching the conclusion that the labrum is responsible for the patient’s pain.

Similarly, Schwartzberg has shown that MRI documented SLAP lesions is present in 55-72% of the asymptomatic middle age population [15]. Outlet impingement, tendinosis/tendonitis, subacromial bursitis and acromioclavicular arthritis are all common pain generators in this demographic population, and focus on the SLAP lesion itself may be misdirected. In 2005, an MRI analysis of professional handball players demonstrated abnormalities in 93% of shoulders with only 37% being symptomatic [16]. A detailed history, physical exam, and correlation of imaging including MRI and or diagnostic and therapeutic injection findings are mandatory before concluding that the SLAP lesion is the patient’s source of pain, especially in this age group.

It is important to realize that not all SLAP lesions require surgery, and for those that do, not all patients benefit from the same surgery. The key is to determine whether the labrum is solely responsible for the patient’s symptoms and whether restoring the attachment of the labrum and biceps root to the glenoid will be helpful. In the older patient with or without rotator cuff repair, the repair of the SLAP is associated with inferior results in comparison to intentional neglect or performing a bicep tenodesis/tenotomy in regards to stiffness, persistent pain and need for revision. A number of authors report good results in athletes, including those with moderate overhead usage (recreational/competitive tennis and softball, swimming, *etc*.) [17]. However, in high-level overhead throwers, several studies have reported a relatively low level of success, and our own data indicate that in professional baseball players, surgical repair has demonstrated poor results as measured by successful return to the previous level of competitive success. Superior outcomes have been demonstrated with non-surgical treatment [14, 17-19].

The best treatment for Type 2 SLAP tears in the overhead athlete is now more controversial than in the past when the surgical repair was routinely performed. While repair of Type 2 SLAP lesions was 9.4% of all applicants’ shoulder procedures performed by ABOS Part 2 candidates from 2003 to 2008 [20], the rate has been diminishing as results of clinical studies indicate that success rates in overhead athletes and in middle-aged patients are lower than expected [19, 21, 22]. Alternative surgical treatments such as bicep tenodesis or tenotomy for the middle-aged population and those with rotator cuff tears have emerged as options preferred by some authors [23, 24] and may be applicable for younger athletes as well [25-27]. Others recommend simple debridement of unstable labral tissue in overhead athletes. However, in overhand throwers, surgery is often not necessary if a focused rehabilitation program dedicated to stretching the posterior capsule and improving scapular dynamics is completed.

## 2 MATERIALS AND METHODS

We evaluated the injury history of 1750 high level collegiate and high school baseball players eligible for the Major League Baseball draft over a three-year period. Fifty-two pitchers were signed, including free agents. Every pitcher underwent an in-person evaluation and MRI scan of the shoulder at intake. The MRI evidence of labral lesions and rotator cuff pathology was assessed. The pre-signing history and subsequent injury history for all players were documented.

## 3 RESULTS

Of the 1750 players (all positions) with reviewed available pre-draft histories, a history of MRI documented SLAP lesion was found in 17%. Not all MRI had been done for shoulder pain, some had been done as screening. Those that returned to play with sufficient proficiency were considered for the draft. The rate of injury following matriculation as a professional baseball pitcher (N=52) in our organization was followed. In three years of professional baseball, 1 player (1.9%) required surgery for a failed previously repaired SLAP lesion and none for a new injury.

## 4 DISCUSSION

The assessment of shoulder injuries in athletes and the general population is challenging particularly in the overhead throwing athlete. Reliance on MRI scans will over-diagnose labral pathology and lead to excessive or unnecessary treatment. Clinical evaluation is of critical importance to accurately diagnose and successfully treat shoulder injuries while avoiding unnecessary or potentially harmful surgery. The above pre-draft assessments should not be construed as a reliable prevalence statistic since the MRI was done for a variety of reasons including injuries in other sports, screening by concerned parents/agents, and was self-reported by players hoping to be drafted. However, the rate of shoulder surgeries for SLAP lesions is indicative of the relatively low probability of operative intervention being required if proper diagnosis and non-surgical treatment are rendered.

### 4.1 Clinical Evaluation

#### 4.1.1 Throwers

In our population of professional overhead throwers, complaints of shoulder pain are investigated with a thorough history and physical exam. As we expect an MRI to demonstrate labral changes regardless of the cause of the player’s pain, MRIs are reserved for recalcitrant cases. Key aspects of the patient’s history are the locations of pain and the provocative phase or phases of pitching. Appreciating the stress on anatomic structures during the phases of pitching facilitates the diagnosis. In our experience, posterior pain with late cocking usually indicates a posterior superior labral tear or hypertrophy with or without undersurface tearing of the infraspinatus-supraspinatus junction. This is commonly referred to as “internal impingement” and is often caused by a flap of tissue (either labral or cuff) being trapped in the cocked position. Posterior pain during release or follow through is most commonly indicative of an eccentric failure of the posterior rotator cuff. Anterior pain during cocking is typically associated with some degree of dynamic anterior instability which can be multifactorial (cuff weakness, scapular dyskinesia, capsular laxity, labral pathology, etc.). Anterior pain with “finishing” the throw or terminal stage of follow-through can indicate mechanical impingement of the bicep or lesser tuberosity on the coracoid. These must all be questioned during the history, correlated with a provocative physical exam and critically evaluated with imaging, especially the MRI.

**Fig. (1):**
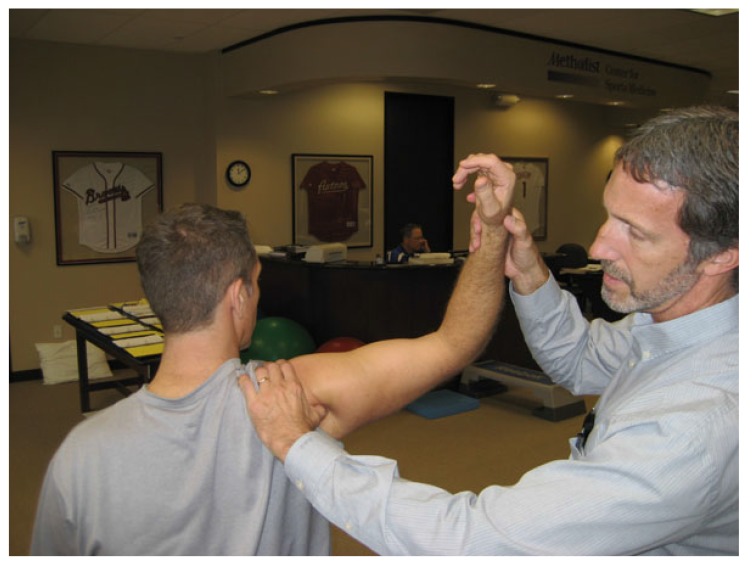
Clinical photograph demonstrating positioning of the Internal Impingement Test. The patient must delineate the location and character of pain (pinching, catching, and instability). Localized pain in the posterior joint line is suggestive of internal impingement.

**Fig. (2):**
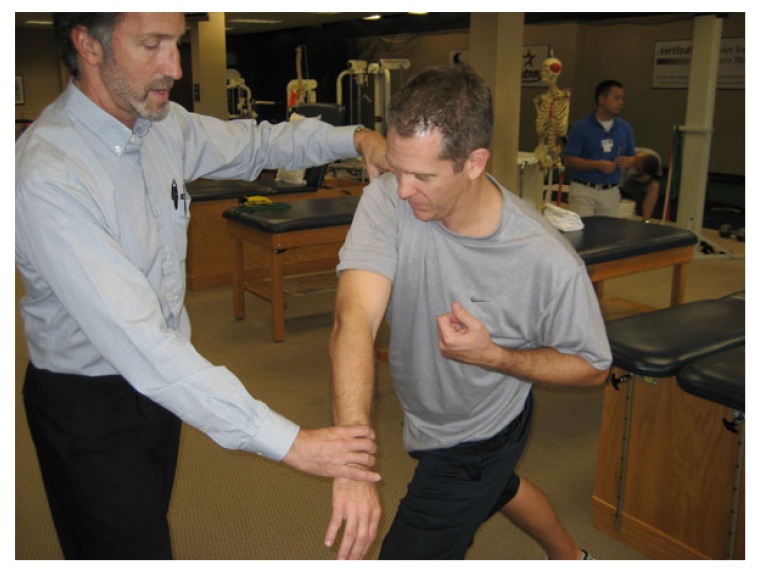
Clinical photograph demonstrating position to assess for a Deceleration sign. With the patient in the follow through position of pitching, posterior pain with applied downward pressure of the wrist is suggestive of inflammation potentially due to eccentric failure or excessive strain of the posterior rotator cuff.

**Fig. (3):**
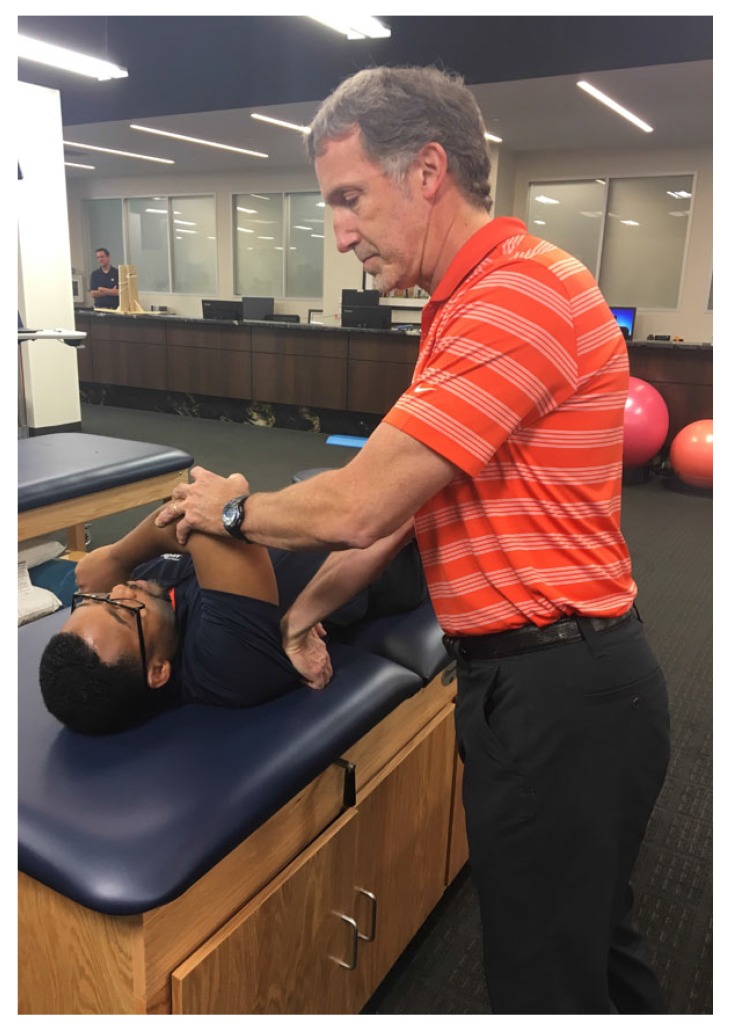
Clinical photograph demonstrating positioning to assess for Posterior Capsule Tightness. The forearm must be kept in neutral rotation to adequately access the posterior inferior capsule. An inability of the elbow to cross the midsagittal plane is suggestive of tight posterior capsule.

On physical exam, special attention to labral signs is helpful, with the O’Brien sign and Jobe Relocation test. Less commonly performed tests can be helpful in the throwing population such as the “Internal Impingement Sign” which is positive when posterior pain is reproduced by exaggerating the cocked position, especially with horizontal extension in the 90 degrees abducted position. Reproducing the patient’s localized pain in the posterior joint line during this maneuver indicates mechanical entrapment of posterosuperior labrum, rotator cuff tissue or both. This maneuver is similar to performing apprehension testing in the standing position but with increased horizontal extension. The patient must not only identify if this position is painful or uncomfortable, but specifically where the discomfort is located as well as the character of the pain (pinching/catching versus pain or apprehension) (Fig. **1**). The deceleration sign is positive when the eccentric contraction of the humeral decelerators is painful in the follow through position. This is tested by placing the patient in the follow through position of throwing and applying downward pressure to the wrist while the patient resists the applied force. Posterior pain is suggestive of inflammation and eccentric failure of the posterior rotator cuff (Fig. **2**). Scapula Dyskinesia and scapular posture can be assessed by inspecting scapular kinesis from behind with the patient moving his arm through all planes including internal and external rotation in the 90-degree abduction position. Thorough evaluation of winging, relative protrusion, and fluid movements of glenohumeral motion with relation to scapulothoracic motion. Hitches, sudden jump/catches, or pain should be assessed with shoulder flexion. Tightness of the posterior capsule is assessed with the patient supine and the examiner immobilizing the scapula against the table. The examiner pushes the humerus across the chest while stabilizing the lateral border of the scapula and keeping the forearm in neutral rotation to prevent coupled external rotation. The shoulder will externally rotate as the arm is pushed into adduction as the posterior-inferior capsule comes under tension. This must be resisted by the examiner in order to obtain accurate and reproducible results. A tight capsule will prevent the elbow from passing through the midsagittal plane, while a flexible capsule will allow movement almost to the midline. Reproduction of pain with the supine cross body test is indicative of posterior capsule tightness as the pain generator (Fig. **3**).

#### 4.1.2 Non-throwers

The evaluation in non-overhead throwing athletes is similar to an overhead thrower, but the positional tests specific to throwing are deferred. The strength of the rotator cuff is evaluated in multiple planes, and signs specific to the long head bicep tendon are prioritized. Reproduction of symptoms with rotator cuff tests, impingement signs, and bicep tests is important. Specifically checking for coracoid impingement in those with anterior pain can be illuminating. The critical assessment of the posterior capsule and potential cuff contracture is imperative especially with the shoulder in 90 degrees of abduction. Comparisons between the symptomatic extremity relative to the asymptomatic side aid in determining the baseline from symptomatic pathology.

### 4.2 Confounding Issues

Those with symptomatic SLAP lesions typically present with a history identical to those with overuse, inflammatory, or rotator cuff pathologies. A thorough assessment through history and physical exam to differentiate between them is imperative, and the physician must not rely solely on MRI imaging. Common alternative diagnoses must also be considered, such as Glenohumeral Internal Rotation Deficit (GIRD), posterior capsule inflammation, adhesive capsulitis, subacromial/subcoracoid bursitis, bicep tendonitis and rotator cuff tendonitis. In addition. multiple varieties of mechanical impingements (outlet, coracoid, posterior) may cause pain. The patient’s activities, location of pain, age, and degree of impairment are all helpful clues to an adequate differential diagnosis.

We have found that the physical exam is the most helpful way to differentiate and attain the correct diagnosis. The most helpful findings on history and physical exam to differentiate between the pain of SLAP origin versus rotator cuff functional failure are location of pain during the phase of throwing and provocative signs on exam, especially after throwing. The local anesthetic injection can be helpful to confirm a subacromial source of symptoms. However, an intra-articular injection may prompt a relief of pain but does not differentiate between mechanical entrapment of tissue, capsulitis, bicep tendonitis, dynamic instability due to cuff failure, or instability due to SLAP lesion as the source of that pain.

We have a full-size pitching lane in our contiguous physical therapy clinic which allows examination immediately after throwing. The most common combination of history and examination findings indicating the root cause of symptoms is demonstrated in Table **1**. Although they are not universal, they provide a reliable rationale for treatment programs. Note that in all cases, the presence of an MRI documented Type 2 SLAP is expected but often irrelevant.

### 4.3 Decision Making

#### 4.3.1 Treatment in Throwers

Our data has demonstrated that surgical treatment of SLAP lesions in throwers has a significantly lower success rate compared to directed rehabilitation that prioritizes the posterior capsule flexibility and scapula positioning. Up to 85% of patients with isolated SLAP tears treated with non-operative management have done well. While many authors have reported good results with surgical repair [18, 21, 28-30], the criteria used do not accurately reflect on-the-field performance. Rather than, examining traditional subjective outcome measures or physical findings such as range of motion or strength, we studied the actual competitive performance of professional pitchers who had MRI documented SLAP lesions that failed a course of Physical Therapy (PT) and underwent surgical SLAP repair. When true baseball performance metrics were applied to pitchers, for those that underwent surgery, the return to play rate was 48% with a return to prior level of play rate at 7% compared to 40% with a return to play rate and a return to prior level of play at 22% rate for those who participated in physical therapy with a focus on cross body stretch and scapular posture [19]. Since that study was completed, we have noted an even higher success rate with non-operative management. We have found that in our overhead athletes, prognostic indicators of successful non-surgical management are a tight posterior capsule with a stretch that reproduces the pain and scapular dyskinesis with a forward rotated and protracted posture.

The role of posterior capsule and posterior rotator cuff tightness in the painful thrower’s shoulder has been well described [31-33]. This can be evaluated through the assessment of Glenohumeral Internal Rotation Deficit (GIRD) which is measured with the arm in 90 degrees of shoulder abduction and 90 degrees of elbow flexion (90-90 position) with comparisons of each side with internal and external rotation. This assessment provides an easy and reproducible measure of capsular maladaptation in this direction. Whether a portion of the loss of internal rotation is caused by bony retroversion is irrelevant since in practicality the bony component cannot be corrected. Wilk et at showed that within 122 professional baseball players the total arc of motion averaged 193 degrees with 130 degrees of external rotation and 63 degrees of internal rotation [34]. Of the players that demonstrate GIRD, they were twice as likely to get injured in a three-year window [35]. Posterior capsule and rotator cuff tightness is most commonly treated with internal rotation stretching at 90 degrees of shoulder abduction in a sleeper stretch and passive internal rotation stretching at 90 degrees of shoulder abduction. Other adaptations have provided improved results in the correction. McClure et al demonstrated that self-applied seated cross-body stretch with internal rotation and no scapular stabilization provided greater internal rotation [36]. Muscle energy techniques have also demonstrated greater immediate effects especially in cross body and internal rotation. Moore et al demonstrated in division 1 college pitchers that resisted contractions and relaxation of three reps in crossbody with internal rotation is more effective than similar techniques applied in internal rotation [37]. The stretching protocol can be a long arduous process, as pitchers involved with these stretches for a prolong period of time of three or more years have shown a greater total arc of the dominant side compared to their non-dominant side. For pitchers that have participated in these stretches for less than three years, the total arc of motion was almost equivocal [38]. Despite these common stretches, the mid-posterior capsule can remain tight even in those who enrolled in a stretching program and demonstrate improved internal rotation at 90 degrees. While certainly a step in the right direction, we have found the sleeper stretch and the self-administered cross-body stretch to be insufficient in the overhead athlete population. There is a subset of patients whose posterior capsule remains tight in cross-body adduction despite improvement in sleeper stretch internal rotation. If not specifically inspected, this is easily overlooked. These patients will note that this specific stretch of the posterior capsule is painful and, most importantly, reproduces their symptoms.

Patients participating in an internal rotation stretching program have found stretching with their hand behind the back similar to the “Towel Stretch” commonly performed during post-surgical rehabilitation. We do not recommend this stretch or motion for throwers as it promotes a forward rotated and protracted scapular position which increases stress of the anterior-superior capsule.

Scapular malposition of anterior tilt, protraction and upward rotation is commonly seen in overhead athletes [39]. Scapular malposition at rest has also been demonstrated to result in glenoid anterior tilting creating an increased contact in the posterior shoulder due to the increased hyperangulation [40]. Closed chain exercises have been proven to correct scapular malposition in protraction, retraction, elevation and depression [41]. In addition to correct scapular posture, focus on strength and conditioning of periscapular muscles are essential to the stamina and proper mechanics of pitching. In professional baseball pitchers, it has been shown that as scapular musculature fatigues, scapular position increases with anterior tilt and protraction resulting in decreased internal rotation [42, 43].

In throwers with scapular dyskinesia, a simple in-office demonstration can convince the patient that scapula posture control is important for success with non-operative management. This can be demonstrated by starting the patient in their baseline standing position and bringing the arm into a maximally cocked position while holding the scapula in the protracted posture causing the patient to feel the significant strain on their elbow and shoulder. The patient is then later instructed to “Set” their scapula with retraction which allows them to bring their arm further into a cocked position with ease and a lack of discomfort. With the scapula retracted, the shoulder and elbow experience significantly less strain which is immediately apparent to the patient. This demonstration often fosters greater patient commitment towards non-operative management and deters those who may have been biased toward surgical intervention for their observed MRI findings rather than their actual functional pathology.

Rehabilitation is best performed by a physical therapist or certified athletic trainer well-versed in these concepts and baseball throwing mechanics. Assessment of mechanics by a qualified professional is critical to confirm that the player maintains adequate scapular posture while throwing. Having a throwing lane in the therapy clinic is also helpful for observation and analysis of pitching mechanics.

Although posterior stretching has been demonstrated to be successful in 66.7 to 90% of athletes [13], in the cases of patients that have addressed their mechanics, posterior capsule flexibility, and scapular posture, yet fail to successfully return to competition due to continued symptoms, surgical intervention may be necessary. In cases of internal impingement secondary to a hypertrophic labrum or small partial articular-sided cuff tear, debridement of rotator cuff and/or labral flaps is imperative and may be sufficient alone. During the 2017 AOSSM annual meeting Sugaya *et al* demonstrated that overhead athletes with stable Type II SLAP lesions and concomitant PASTA lesions did well with debridement of the posterosuperior labrum and PASTA lesion. For overhead athletes with extensive detachment of the anterior and posterior portion of the labrum, the anterior superior labrum was repaired while the posterosuperior labrum was debrided. Meantime and return rate to play were similar between the debridement only group and the repair group [44, 45]. Debridement of the typically bulky posterior labrum minimizes posterior impingement when cocked. When evaluating softball and baseball players, Ciccotti et al demonstrated that 79.5% of their athletes were able to return to their prior level of activity after surgery. Of these players 84% of pitchers and 72% of the position players were able to return to play [17]. For the case of isolated tight posterior capsules, posterior inferior capsular release has shown to be effective in returning 11 of 16 throwers to their pre-injury level of participation. Two of the 16 had a reduction in pain while the other 14 of 16 had an elimination of pain [46]. Yung *et al* followed 13 overhead athletes with SLAP repairs for 28 months and found that only 4 were able to return to prior levels at 11 months, while 1 never did [47]. In a systematic review which included 506 athletes, 198 overhead athletes, 81 of which were pitchers, 83% of all athletes reported good to excellent satisfaction rates and a return to previous level of play rate at 73%. When looking at only overhead athletes 74% expressed satisfaction of good to excellent, while only 63% percent were able to return to prior levels of play [48]. Many other studies have been published demonstrating a wide array of players that able to return back to prior levels. The return to play rates in overhead athletes have ranged from 22% to 71% with satisfaction levels being reported at around 69% [18, 25, 28, 49, 50]. When addressing unstable Type 2 SLAP tears, the surgeon must be cautious because it has been shown that placing an anchor anterior to the bicep anchor during SLAP repairs can results in decreased external rotation [51] which correlates with a failure to return to competition [52]. In 2011 Paletta *et al* demonstrated that all eight of their pitchers that lost greater than 10 degrees of external rotation at 90 degrees of shoulder abduction were unable to return to throwing [52]. Due to these findings, it is advocated that the labrum should be repaired only behind the bicep anchor in this population to prevent the loss of external rotation. In regards to management of the long head of the biceps tendon, primary bicep tenodesis is being increasingly chosen as a treatment of choice versus slap repair [53]. With the exception of Type 4 SLAP lesions, bicep tenodesis is not recommended in the young thrower population as the long head of the bicep tendon is thought to be a dynamic stabilizer and the placement of a drill hole in the proximal humerus creates a stress riser in an area subject to high torsional stress. In the senior author’s (DML) experience, surgery on the long head of the bicep tendon as treatment for SLAP induced pain is rarely necessary in this population. The number of tenodeses in professional pitchers is almost nonexistent (as per personal communication and MLB Team Physicians Association Meeting of 2016) due to the concern for stress risers. Tenotomy may be considered if the surgeon is convinced that the bicep tendon itself is the source of discomfort. However, routine tenodesis for treatment of shoulder pain in throwers with SLAP lesions noted on MRI would be gross overtreatment in the large majority of cases.

Once the labrum has healed, the principles applied during the non-operative program are again applied postoperatively. During the early post-operative phase, scapular posture is again reinforced and early passive external rotation in the scapular plane is instituted to avoid the critical loss of external rotation. Closed chain cuff exercises using “Ball on the Wall” or Dynamic Stabilization and Strengthening (DS2) platforms are safe and effective modalities to initiate early rotator cuff and scapula stabilization and strengthening.

#### 4.3.2 Treatment of Non-throwers

Non-operative management is the standard for non-throwers as well. Focus on rotator cuff strengthening, scapular kinetics, and capsule flexibility is paramount to recovery. Determining the cause or etiology of the onset of symptoms either through the history or a physical exam can guide the target of the therapy. The physician, physical therapist, or athletic trainer must be cautious with increased or new activities as they can result in painful inflammation that impairs rotator cuff strength resulting in dynamic instability. If overuse induced inflammation is the primary cause of symptoms, anti-inflammatory modalities, medication such as non-steroidal anti-inflammatories or even steroids, and treatment as simple as cryotherapy geared to controlling inflammation are initiated followed by strengthening and training of scapular rhythm. Trauma such as a fall or motor vehicle accident can result in dysfunction of the rotator cuff resulting in a similar cascade, however, treatment should focus more on cuff strength and scapula kinetics in the early phase.

## CONCLUSION

In most cases, an MRI diagnosis of a SLAP lesion is an incidental finding which can cause significant concern in patients leading to unnecessary and sometimes harmful surgical intervention. In order to avoid patient anxiety and overtreatment, it is important to educate patients about the prevalence of asymptomatic SLAP lesions in the general population before an MRI is performed. Once diagnosed, it is highly recommended that the patient understands that the labrum is only one of a number of issues (posterior capsule, scapula dyskinesia, *etc*.) in the shoulder and that a concerted non-operative protocol with focus on scapular posture, strengthening and stretching with a well-qualified physical therapist or trainer has a high likelihood of success prior to considering any surgical intervention. If an adequate attempt at non-operative management fails (as defined by improvement in posterior capsule tightness and scapula posture), surgical intervention must be carefully weighed with care, with an understanding that the prior level of performance in overhead athletes may not be achieved. Combining tenodesis with SLAP repair in throwers is not advised due to the expected loss of external rotation as well as the creation of a stress riser in the proximal humerus. In the non-throwing athlete, pain relief is usually substantial and the risks acceptable. In the non-athlete with tenodesis without SLAP repair, pain relief is significant with few complications.

## Figures and Tables

**Table 1:** Features of the history and physical exam in the painful thrower’s shoulder.

**Source of Pain**	**Phase**	**Pain Location**	**Symptom Reproduction**
**Mechanical entrapment of labrum or cuff fragment**	Cocking	Posterior	Posterior impingement sign
**Cuff failure**	Release through follow thru	Anterior	Reproduced pain with eccentric cuff stress in follow thru position
**Posterior Capsule**	Any	Deep posterior	Reproduced with cross body stretch
**Subacromial**	Any	Superior/lateral, posterior	Positive Impingement signs Positive Impingement test

## LIST OF ABBREVIATIONS

DS2: Dynamic Stabilization and Strengthening
GIRD: Glenohumeral Internal Rotation Deficit
MRI: Magnetic Resonance Imaging
PT: Physical Therapy
SLAP: Superior Labral Anterior to Posterior
